# 1580. A national prospective HIV provider survey of antiretroviral therapy preferences for management of treatment naïve and experienced individuals with genotypic resistance

**DOI:** 10.1093/ofid/ofad500.1415

**Published:** 2023-11-27

**Authors:** Sonya Krishnan, Stephanie Bjerrum, Marina B Martinez Rivera, Maunank Shah

**Affiliations:** Johns Hopkins University School of Medicine, Baltimore, Maryland; Rigshospitalet, Department of Infectious Diseases, Koebenhavn, Hovedstaden, Denmark; Johns Hopkins University, Baltimore, Maryland; Johns Hopkins, Baltimore, MD

## Abstract

**Background:**

HIV clinical practice guidelines outline broad treatment principles, but offer less explicit recommendations for complex patient situations. We hypothesize there is variability in antiretroviral (ARV) decision-making among experienced providers when considering HIV drug resistance.

**Methods:**

US HIV clinicians and pharmacists were enrolled to provide ARV recommendations for up to 36 clinical case-vignettes in a series of electronic surveys (minimum survey length, 6 cases), encompassing variations of ARV resistance. Responses were categorized for each case based on drugs and classes selected, as well as anticipated activity based on genotypic susceptibility.

**Results:**

119 experienced clinicians from across the US participated (Table 1). In the setting of high-level viremia and an isolated M184V, 85.9% selected a regimen with 2 NRTI+INSTI, while 9.9% selected regimens with > 3 ARVs (Table 2, Case #1). Alternatively, when presented scenarios with treatment-failure and moderate to high-level NRTI resistance (Table 2, Case #2, 3) without PI or INSTI resistance, providers most frequently ( >50%) selected an NRTI-sparing regimen, while a minority recommended 2NRTI + INSTI (21/123). Following EVG/c/TAF/FTC failure (isolated M184V, potential low level INSTI resistance, Table 2, Case #4), most providers recommended a PI-based regimen (31.2%) with 2 NRTI, or an intensified regimen with > 3 drugs (32.5%), while a minority suggested a second generation INSTI (23.4%). By contrast, when low-level INSTI resistance was present with more extensive NRTI resistance (Table 2, Case #5), the majority chose an NRTI-sparing regimen (45.6%) or a regimen with > 3 drugs (e.g., 2NRTI+INSTI+PI; 36.8%).

**Table 1.**
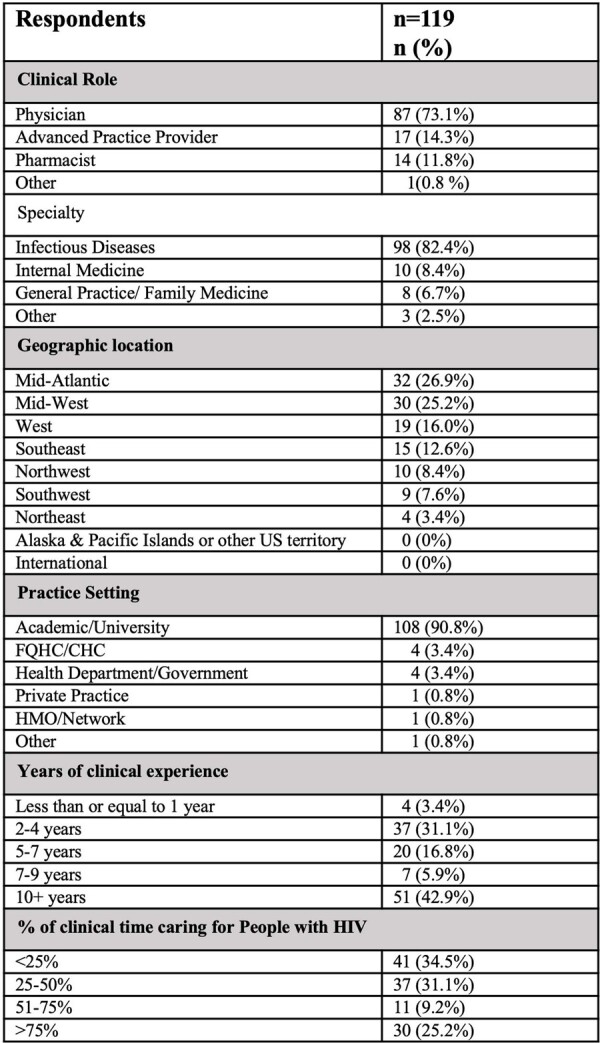
Characteristics of survey respondents

**Figure:**
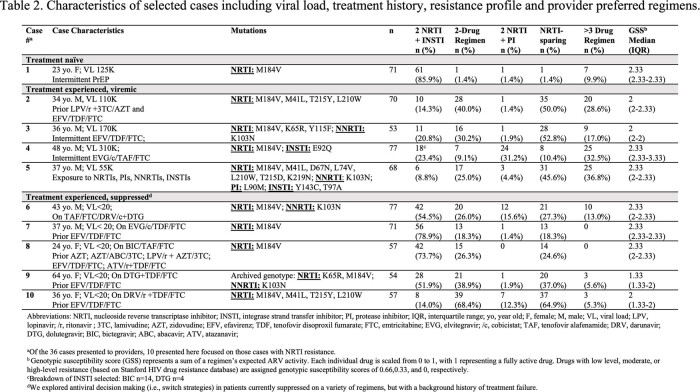

**Conclusion:**

There was heterogeneity in treatment preferences among providers when presented with ARV resistance. A majority suggested INSTI-based regimens (with 2 NRTI) with limited NRTI resistance alone. By contrast, most providers recommended regimens with at least 2 active drugs (e.g., NRTI-sparing or intensified with PI and INSTI) with more extensive NRTI resistance.

**Disclosures:**

**All Authors**: No reported disclosures

